# Activities of leaf and spike carbohydrate-metabolic and antioxidant enzymes are linked with yield performance in three spring wheat genotypes grown under well-watered and drought conditions

**DOI:** 10.1186/s12870-020-02581-3

**Published:** 2020-08-31

**Authors:** Sajid Shokat, Dominik K. Großkinsky, Thomas Roitsch, Fulai Liu

**Affiliations:** 1 Crop Science, Department of Plant and Environmental Sciences, University of Copenhagen, Højbakkegård Allé 13, 2630 Taastrup, Denmark; 2 Wheat Breeding Group, Plant Breeding and Genetic Division, Nuclear Institute for Agriculture and Biology, Faisalabad, 38000 Pakistan; 3 Transport Biology, Department of Plant and Environmental Sciences, Copenhagen Plant Science Centre, Thorvaldsensvej 40, 1871 Frederiksberg C, Denmark; 4 AIT Austrian Institute of Technology GmbH, Center for Health and Bioresources, Bioresources Unit, Konrad-Lorenz-Straße 24, 3430 Tulln, Austria

**Keywords:** Antioxidant activity, Carbohydrate metabolism, Drought, Kernel abortion, Wheat

## Abstract

**Background:**

To improve our understanding about the physiological mechanism of grain yield reduction at anthesis, three spring wheat genotypes [L_1_ (advanced line), L_2_ (Vorobey) and L_3_ (Punjab-11)] having contrasting yield potential under drought in field were investigated under controlled greenhouse conditions, drought stress was imposed at anthesis stage by withholding irrigation until all plant available water was depleted, while well-watered control plants were kept at 95% pot water holding capacity.

**Results:**

Compared to genotype L_1_ and L_2_, pronounced decrease in grain number (NGS), grain yield (GY) and harvest index (HI) were found in genotype L_3_, mainly due to its greater kernel abortion (KA) under drought. A significant positive correlation of leaf monodehydroascorbate reductase (MDHAR) with both NGS and HI was observed. In contrast, significant negative correlations of glutathione S-transferase (GST) and vacuolar invertase (vacInv) both within source and sink were found with NGS and HI. Likewise, a significant negative correlation of leaf abscisic acid (ABA) with NGS was noticed. Moreover, leaf aldolase and cell wall peroxidase (cwPOX) activities were significantly and positively associated with thousand kernel weight (TKW).

**Conclusion:**

Distinct physiological markers correlating with yield traits and higher activity of leaf aldolase and cwPOX may be chosen as predictive biomarkers for higher TKW. Also, higher activity of MDHAR within the leaf can be selected as a predictive biomarker for higher NGS in wheat under drought. Whereas, lower activity of vacInv and GST both within leaf and spike can be selected as biomarkers for higher NGS and HI. The results highlighted the role of antioxidant and carbohydrate-metabolic enzymes in the modulation of source-sink balance in wheat crops, which could be used as bio-signatures for breeding and selection of drought-resilient wheat genotypes for a future drier climate.

## Background

Cultivation of wheat in an ever-decreasing water scenario has posed enormous challenges to meet the global food security. Water is vital throughout wheat growth phases however anthesis and post-anthesis stages are considered more sensitive to drought [1–3]. Limited water availability at these stages directly affects grain number and grain weight leading to severe reduction in yield potential [1]. In the past, this crop has been improved [4] however, there is a need to understand the in-depth physiological mechanism to breed drought-resilient wheat. Primarily, plant drought avoidance is achieved through closure of stomata. This closure of stomata is induced mainly through abscisic acid (ABA) which is transported from roots to leaves via xylem vessels to induce stomatal closure [5]. Plant genotypes having high yield potential under drought often regulate their stomata to maintain higher photosynthetic rate while lowering transpiration rate thus an enhancement of water use efficiency results in a less reduction of biomass and grain yield [6, 7].

It is well-known that reduced photosynthesis under drought is associated with modified carbohydrate metabolism of leaves [8, 9]. On the other hand, reduction of sink strength due to impaired carbohydrate metabolism has been reported in reproductive tissues under drought stress [10]. Under drought, limited supply of carbon creates a competition between sink organs, which results in reduced sink strength and yield [11]. Several key metabolic enzymes have been reported for their vital roles in sugar conversion within source and sink. These enzymes have also been demonstrated for their striking roles in abiotic stress tolerance as well. Albacete et al. [12] found that plant adaptation to drought stress can be improved by the over-expression of a cell wall invertase gene (CIN1). Under severe drought stress conditions, when very little or no photosynthates are being synthesized or translocated to the developing sinks, a decrease in the activity of cell wall invertase (cwInv) enzyme could happen [12], and plants may turn to use the stored carbohydrates to fulfil their energy demands [13, 14]. This response may be associated with an increased activity of other invertases like cytoplasmic (cytInv) and vacuolar invertase (vacInv), which facilities the remobilization of photo-assimilates from source to sink [15]. VacInv plays a role in sugar storage and higher vacInv activity stimulates the remobilization of stored carbohydrates into reproductive organs, is required for normal plant growth [16]. Higher vacInv activity was associated to faster development of cotton fibers by contributing to cell expansion [17]. Over-expression of vacInv increased stomatal opening in cotton, indicating role of vacInv in stomatal regulation [18]. In *Arabidopsis thaliana*, the osmotic potential has been reported to be regulated through an increase in vacInv activity both under drought and salinity stress [19]. Similarly, ABA-induced increase in vacInv activity contribute towards hexose accumulation hence osmotic adjustment in maize leaves [20]. The growth of *A. thaliana* was not affected by deficiency in sucrose synthase (SuSy) but was severely reduced due to deficiency in cytInv, indicating a more important role for cytInv in coordinating the metabolism [21]. Aldolase is an important enzyme of glycolysis playing an important role in plant development, regulation and biotic and abiotic stresses tolerance [22]. A decrease in aldolase activity has been found in chickpea under drought stress [23], while the overexpression of aldolase increased photosynthetic rate, growth and biomass in tobacco [24].

Carbohydrate partitioning depends upon the sink strength, which is altered under water deficit [25]. The transport of carbohydrates from source to sink is required for the development of grains. Massive kernel abortion and limited grain filling due to a slight decrease of soluble sugars within source tissue has been observed in maize [26]. Likewise the activity of adenosine diphosphate-glucose pyrophosphorylase (AGPase) is associated with grain filling [27]. Under drought stress, a positive correlation of AGPase with developing grains is reported in wheat and rice [15] [28], while a decrease in AGPase activity was reported in sensitive genotypes of wheat [29].

Thus, the modulation of the carbohydrate-catalyzing enzymes activity in response to drought in both source and sink organs of crop plants would play a crucial role in determining reproductive development and yield. In the past, the role of individual enzymes had been investigated by many researchers both under well-watered and drought conditions [12, 19, 30]. However, the global correlations between the yield components and carbohydrate metabolism in source and sink of wheat as affected by drought stress remains largely unknown and merits further investigations.

Drought stress also induces the accumulation of reactive oxygen species (ROS) within plant cells [31]. Plants detoxify ROS through enzymatic and non-enzymatic antioxidants. First line of abiotic stress tolerance includes superoxide dismutase (SOD), catalase (CAT), peroxidases (POX), and different studies reported a steady-level of CAT [32] while enhanced activity of SOD and POX [33–35] under stress conditions. Likewise, other antioxidant enzymes of the ascorbate-glutathione cycle, such as monodehydroascorbate reductase (MDHAR), dehydroascorbate reductase (DHAR) and glutathione reductase (GR) are important to maintain the redox homeostasis under abiotic stress [31, 36]. Studies reported an increase in the activity of MDHAR in rice under drought stress and enhanced activity of DHAR as well as GR under drought stress in wheat [33, 35, 36]. Moreover, glutathione-S-transferase (GST) also plays an important role to reduce the oxidative damage within plants [37, 38] to improve the tolerance to different stresses [38]. Plant genotypes having higher activity of these antioxidants are expected to produce more yield under stress conditions. Studies have been conducted to understand the role of these antioxidants during drought stress in wheat [33, 35], however, their role in relation to carbohydrate metabolic enzymes could explain the mechanism of drought tolerance in depth.

In this study, the response of three wheat genotypes namely L_1_ (advanced line), L_2_ (Vorobey) and L_3_ (Punjab-11) having contrasting yield potential under drought stress was studied under controlled greenhouse condition by imposing drought stress at anthesis stage in pot experiment. Our aim was to explore the variation in leaf, spike carbohydrate metabolic and antioxidant enzyme activity signatures as well as their associations with yield and its attributes under well-watered and drought stress conditions. The results will help to find discriminating biomarkers in order to devise future strategies for breeding drought resilient wheat cultivars.

## Results

### Leaf gas exchange and water relations

No significant differences in stomatal conductance (Gs), photosynthetic rate (An), relative water content (RWC) and osmotic potential (Ψ_π_) were observed between the three genotypes. Compared to the well-watered controls, drought stress significantly decreased Gs, An, RWC and Ψ_π_ in all genotypes (Table 1). Genotypes were significantly different for osmotic adjustment (OA) and highest value of OA was recorded in genotype L_2_ while lowest was in L_1_ (Table 2).

**Table 1:** Analysis of variance, mean and standard errors of the three genotypes for photosynthesis, stomatal conductance and transpiration rate under well-watered and drought conditions

Genotype	Photosynthesis (An, μmol m^−2^ s^− 1^)		Stomatal conductance (Gs, mol m^− 2^ s^− 1^)		Transpiration rate (E, mmol m^− 2^ s^− 1^)	
	WW	D	WW	D	WW	D
**L**_**1**_	17.4 ± 1	−1.63 ± 0.18	0.32 ± 0.06	0.03 ± 0.01	4.32 ± 0.55	0.39 ± 0.05
**L**_**2**_	18.88 ± 1.61	0.63 ± 0.16	0.32 ± 0.08	0.07 ± 0.03	2.992 ± 0.20	0.3206 ± 0.08
**L**_**3**_	16.84 ± 1.69	−1.17 ± 0.09	0.39 ± 0.05	0.02 ± 0.01	5.06 ± 0.48	0.4352 ± 0.07
**P**_**G**_	0.13		0.83		0.002	
**P**_**W**_	< 0.001		< 0.001		< 0.001	
**P**_**WxG**_	0.87		0.45		0.005	

***WW*** Well-watered, ***D*** Drought, ***P***_***W***_**,** *P* value of drought effect, ***P***_***G***_ *P* value of genotype effect and ***P***_***WxG***_ *P* value of the interaction of drought by genotype

**Table 2:** Analysis of variance, mean and standard errors for plant water relations of the three genotypes of wheat under well-watered and drought conditions

Genotypes	L_1_		L_2_		L_3_		*P* value
	WW	D	WW	D	WW	D	
Relative water content (%)	91.78 ± 1.54	53.35 ± 3.89	92.71 ± 1.79	61.28 ± 5.36	94.56 ± 1.33	47.09 ± 4.91	P_G_ = 0.39 P_W_ < 0.001 P _W*G_ = 0.06
Leaf osmotic potential (MPa)	−1.04 ± 0.08	−2.31 ± 0.11	−0.9 ± 0.12	−2.85 ± 0.44	−0.86 ± 0.07	−2.9 ± 0.05	P_G_ = 0.15 P_W_ < 0.001 P _W*G_ = 0.06
Osmotic adjustment (OA)		0.24 ± 0.16		0.96 ± 0.09		0.75 ± 0.13	P_G_ < 0.01

***WW*** well-watered, ***D*** Drought, ***P***_***W***_ *P* value of drought effect, ***P***_***G***_ *P* value of genotype effect and ***P***_***WxG***_ *P* value of the interaction of drought by genotype

### Activities of carbohydrate metabolic enzymes in leaf

Activities of leaf vacuolar invertase (vacInv) was significantly different between the three genotypes under control conditions. The highest activity of vacInv was recorded in L_3_ and lowest in L_1_. Compared to well-watered conditions, no significant differences were recorded for the activity of vacInv under drought stress. All genotypes exhibited similar cytoplasmic invertase (cytInv) activity under control conditions, while drought caused a non-significant increase of cytInv activity. The activity of cwInv was statistically similar among the genotypes, though L_3_ showed a lower activity than L_1_ and L_2_ under control conditions. Drought significantly enhanced the activity of this enzyme in comparison to the well-watered controls (Table 3).

**Table 3:** Analysis of variance, mean and standard errors for the activity of carbohydrate metabolic enzymes (nkat/g FW) within leaf and spike tissue under well-watered and drought conditions

Enzymes activity in leaf							
Genotypes	L_1_		L_2_		L_3_		*P*-value
	WW	D	WW	D	WW	D	
Cell wall invertase (cwInv)	41.61 ± 11.8	79.73 ± 13.3	43.65 ± 14.3	59.27 ± 14.1	18.57 ± 4.83	64.48 ± 21.16	P_G_ = 0.41 P_W_ = 0.009 P _W*G_ = 0.54
Vacuolar invertase (vacInv)	18.12 ± 0.97	38.88 ± 6.25	36.86 ± 9.19	47.67 ± 8.49	63.61 ± 12.3	144.87 ± 67.1	P_G_ = 0.04 P_W_ = 0.12 P _W*G_ = 0.42
Cytoplasmic invertase (cytInv)	13.54 ± 1.96	25.96 ± 9.56	13.69 ± 3.45	22.83 ± 7.48	13.94 ± 1.52	31.07 ± 17.02	P_G_ = 0.88 P_W_ = 0.08 P _W*G_ = 0.89
Adenosine diphosphate phosphorylase (AGPase)	2.82 ± 0.20	2.36 ± 0.30	1.75 ± 0.17	1.34 ± 0.31	2.76 ± 0.27	2.06 ± 0.38	P_G_ = 0.003 P_W_ = 0.03 P _W*G_ = 0.87
Uridine diphosphate glucose phosphorylase (UGPase)	25.10 ± 1.35	22.75 ± 3.32	20.08 ± 1.44	13.65 ± 1.13	22.58 ± 2.34	22.14 ± 1.89	P_G_ = 0.01 P_W_ = 0.13 P _W*G_ = 0.3
Hexokinase (HXK)	0.05 ± 0.01	0.11 ± 0.02	0.20 ± 0.4	0.07 ± 0.03	0.08 ± 0.004	0.15 ± 0.02	P_G_ = 0.08 P_W_ = 0.99 P _W*G_ < 0.001
Fructokinase (FK)	1.46 ± 0.17	0.81 ± 0.15	1.38 ± 0.28	0.97 ± 0.29	2.66 ± 0.20	1.46 ± 0.50	P_G_ = 0.02 P_W_ = 0.2P _W*G_ = 0.75
Phosphoglucomutase (PGM)	13.14 ± 1.90	12.39 ± 2.97	11.87 ± 2.39	8.84 ± 1.93	8.27 ± 0.51	8.40 ± 1.81	P_G_ = 0.11 P_W_ = 0.47 P _W*G_ = 0.72
Phosphoglucoisomerase (PGI)	9.55 ± 0.49	13.34 ± 0.91	15.04 ± 1.93	11.43 ± 1.78	17.62 ± 0.65	21.02 ± 1.69	P_G_ < 0.001 P_W_ = 0.31 P _W*G_ = 0.03
Phosphofructokinase (PFK)	0.41 ± 0.06	0.39 ± 0.09	0.31 ± 0.06	0.19 ± 0.02	0.53 ± 0.3	0.43 ± 0.13	P_G_ = 0.02 P_W_ = 0.20 P _W*G_ = 0.76
Fuctose-1,6-bisphosphate aldolase (Aldolase)	0.30 ± 0.08	0.23 ± 0.15	0.54 ± 0.09	0.11 ± 0.05	0.56 ± 0.03	0.33 ± 0.07	P_G_ = 0.21 P_W_ < 0.001 P _W*G_ = 0.07
**Enzymes activity in spike**							
Genotypes	L_1_		L_2_		L_3_		*P*-value
	WW	D	WW	D	WW	D	
Cell wall invertase (cwInv)	28.73 ± 9.45	21.67 ± 2.52	49.52 ± 19.4	58.19 ± 15.7	49.68 ± 28.4	63.50 ± 20.44	P_G_ = 0.18 P_W_ = 0.73 P _W*G_ = 0.83
Vacuolar invertase (vacInv)	18.12 ± 0.97	47.72 ± 14.2	36.86 ± 9.2	47.67 ± 8.49	92.56 ± 46.7	128.22 ± 40.4	P_G_ = 0.02 P_W_ = 0.25 P _W*G_ = 0.88
Cytoplasmic invertase (cytInv)	64.66 ± 8.79	87.68 ± 11.6	7.73 ± 4.01	34.53 ± 7.85	30.68 ± 6.28	57.22 ± 15.81	P_G_ < 0.001 P_W_ = 0.005 P _W*G_ = 0.98
Adenosine diphosphate phosphorylase (AGPase)	0.43 ± 0.04	0.58 ± 0.05	0.77 ± 0.02	0.87 ± 0.04	0.60 ± 0.11	0.48 ± 0.05	P_G_ < 0.001 P_W_ = 0.37 P _W*G_ = 0.08
Uridine diphosphate glucose Phosphorylase (UGPase)	10.63 ± 0.63	11.49 ± 0.94	9.42 ± 1.09	10.16 ± 1	9.59 ± 0.27	10.46 ± 0.29	P_G_ = 0.23 P_W_ = 0.09 P _W*G_ = 0.93
Hexokinase (HXK)	0.04 ± 0.01	0.09 ± 0.02	0.11 ± 0.01	0.12 ± 0.02	0.08 ± 0.01	0.09 ± 0.01	P_G_ = 0.003 P_W_ = 0.03 P _W*G_ = 0.23
Fructokinase (FK)	0.41 ± 0.08	0.47 ± 0.04	0.56 ± 0.18	0.84 ± 0.1	0.41 ± 0.1	0.56 ± 0.06	P_G_ = 0.04 P_W_ = 0.06 P _W*G_ = 0.56
Phosphoglucomutase (PGM)	5.38 ± 0.74	5.80 ± 0.47	4.32 ± 0.69	5.09 ± 1.03	3.17 ± 0.19	3.71 ± 0.35	P_G_ = 0.01 P_W_ = 0.23 P _W*G_ = 0.96
Phosphoglucoisomerase (PGI)	4.63 ± 0.31	5.99 ± 0.46	7.69 ± 0.64	7.44 ± 0.89	6.05 ± 0.85	6.01 ± 0.99	P_G_ = 0.02 P_W_ = 0.56 P _W*G_ = 0.56
Phosphofructokinase (PFK)	0.22 ± 0.02	0.34 ± 0.03	0.10 ± 0.01	0.25 ± 0.04	0.11 ± 0.04	0.18 ± 0.01	P_G_ < 0.001 P_W_ < 0.001 P _W*G_ = 0.30
Fuctose-1,6-bisphoate aldolase (Aldolase)	0.05 ± 0.01	0.05 ± 0.03	0.13 ± 0.07	0.07 ± 0.02	0.09 ± 0.03	0.05 ± 0.01	P_G_ = 0.08 P_w_ = 0.00 P _W*G_ = 0.56

***WW*** Well-watered, ***D*** Drought, ***P***_***W***_*P* value of drought effect, ***P***_***G***_ *P* value of genotype effect and ***P***_***WxG***_ *P* value of the interaction of drought by genotype

The activities of AGPase and UGPase were significantly different among the three genotypes where, the lowest activities of both enzymes were noticed in L_2_ in comparison to the other two genotypes. Compared to the well-watered controls, significant reduction of leaf AGPase activity by drought was observed. Drought did not affect the activity of UGPase. Also, the activity of fructokinase (FK) was significantly different among genotypes where, higher activity was recorded in genotype L_3_ in relation to the other two genotypes. Drought significantly reduced the activity of FK in comparison to well-watered controls. The activity of hexokinase (HXK) was neither affected by genotype nor by drought; whereas, interaction between water*genotype was significant and prominent increase from 0.2 to 0.07 nkat g^− 1^ Fw observed in genotype L_2_ (Table 3).

Phosphoglucomutase (PGM) activity was statistically similar among the three genotypes Activities of phosphoglucoisomerase (PGI) phosphofructokinase (PFK) varied significantly among three genotypes and highest were recorded in L_3_ in comparison to other two genotypes. Drought did not affect the activity of PGM, PGI and PFK. Non-significant differences were recorded among genotypes for the activity of aldolase. Compared to well-watered controls, activity of aldolase was reduced significantly under drought (Table 3).

### Activities of carbohydrate metabolic enzymes in spike

The activity of vacInv was significantly different among genotypes and the highest activity was recorded in L_3_ in relation to L_1_ and L_2_. Compared to the well-watered controls, the activity of vacInv was not significantly affected by drought. The activity of cytInv enzymes was significantly different among three genotypes where, higher activity was recorded in L_1_ in comparison to other two genotypes. Compared to well-watered controls the activity of cytInv was significantly increased under drought. The activity of cwInv was identical among the three genotypes and it was not affected by drought (Table 3).

Significant differences in the activity of AGPase were found between genotypes and it was highest for L_2_ in comparison to other two genotypes. No significant effect of drought on the activity of AGPase was noticed. Neither genotype nor drought affected the activity of UGPase significantly. The activities of FK and HXK were significantly varied between the genotypes, where higher activities of these enzyme were found in L_2_ compared to other two genotypes, and FK activity was not significantly affected by drought (Table 3). In contrast, the activity of HXK was significantly increased under drought as compared to well-watered control (Table 3).

Differences were significant among genotypes for the activities of PGM and PFK and higher activities were recorded in L_1_ in comparison to other two genotypes (Table 3). Significant differences for the activity of PGI were noticed among the genotypes and higher activity was recorded in L_2_. However, activities of PGM and PGI were not significantly affected by drought. Compared to well-watered controls, drought significantly enhanced the activity PFK. Aldolase activity was neither affected by genotypes nor by drought (Table 3).

### Abscisic acid concentration and antioxidants activities in leaf

Leaf ABA concentrations differed significantly among the three genotypes where highest ABA concentration was recorded in L_2_ compared to other two genotypes. Compared to the well-watered control, leaf ABA concentration was significantly higher under drought conditions. A significant interaction between water*genotype was also noticed for leaf ABA concentration where pronounced effect was recorded in genotype L_3_ (Table 4).

**Table 4:** Analysis of variance, mean and standard errors for the activity of antioxidant enzymes and abscisic acid within leaf and spike under well-watered and drought conditions

Leaf Antioxidants (nkat/g FW)							
Genotypes	L1		L2		L3		*P* value
	WW	D	WW	D	WW	D	
Dehydroascorbate reductase (DHAR)	0.32 ± 0.04	0.75 ± 0.17	0.64 ± 0.22	0.91 ± 0.15	0.59 ± 0.09	0.55 ± 0.12	P_G_ = 0.018 P_W_ = 0.06 P _W*G_ = 0.23
Glutathione reductase (GR)	12.69 ± 1.98	5.28 ± 0.21	4.97 ± 1.41	8.16 ± 5.48	9.03 ± 2.81	9.23 ± 2.01	P_G_ = 0.44 P_W_ < 0.46 P _W*G_ = 0.07
Glutathione-S-transferase (GST)	2.28 ± 0.68	13.47 ± 0.26	3.19 ± 0.26	11.08 ± 0.21	1.49 ± 0.29	10.27 ± 0.2	P_G_ < 0.001 P_W_ < 0.001 P _W*G_ < 0.001
Peroxidase (POX)	9.65 ± 0.49	8.76 ± 2.98	3.08 ± 0.49	2.90 ± 0.42	4.75 ± 1.07	2.91 ± 0.82	P_G_ < 0.001 P_W_ = 0.4 P _W*G_ = 0.83
Cell wall peroxidase (cwPOX)	4.11 ± 0.71	4.28 ± 0.21	5.6 ± 0.32	4.14 ± 0.29	6.46 ± 0.50	4.76 ± 0.19	P_G_ = 0.009 P_W_ = 0.007 P _W*G_ = 0.06
Monodehydroascorbate reductase (MDHAR)	0.28 ± 0.10	0.13 ± 0.04	0.38 ± 0.13	0.22 ± 0.16	0.08 ± 0.03	0.12 ± 0.02	P_G_ = 0.056 P_W_ = 0.17 P _W*G_ = 0.36
Abscisic acid (ng/g FW)	1045 ± 263.8	4776 ± 595	1868 ± 685.5	2062 ± 362.4	428 ± 132.4	3672 ± 933.1	P_G_ < 0.001 P_W_ < 0.001 P _W*G_ < 0.001
**Spike Antioxidants (nkat/g FW)**							
Genotypes	L_1_		L_2_		L_3_		*P* value
	WW	D	WW	D	WW	D	
Dehydroascorbate reductase (DHAR)	0.84 ± 0.03	0.96 ± 0.04	2.12 ± 0.19	1.52 ± 0.12	1.02 ± 0.12	0.91 ± 0.24	P_G_ < 0.001 P_W_ = 0.11 P _W*G_ = 0.06
Glutathione reductase (GR)	3.90 ± 0.68	1.24 ± 0.36	1.14 ± 0.48	2.34 ± 0.67	4.21 ± 0.71	4.09 ± 0.43	P_G_ = 0.002 P_w_ = 0.27 P _W*G_ = 0.01
Glutathione-S-transferase (GST)	2.63 ± 0.67	13.47 ± 0.20	2.24 ± 0.51	11.09 ± 0.34	1.39 ± 0.68	10.28 ± 0.16	P_G_ < 0.001 P_W_ < 0.001 P _W*G_ = 0.07
Peroxidase (POX)	1.87 ± 0.07	1.88 ± 0.09	1.27 ± 0.11	0.91 ± 0.14	3.27 ± 0.38	2.35 ± 0.28	P_G_ < 0.001 P_W_ = 0.67 P _W*G_ = 0.052
Cell wall peroxidase (cwPOX)	3.55 ± 0.17	2.82 ± 0.08	2.60 ± 0.40	2.47 ± 0.31	2.92 ± 0.34	3.31 ± 0.19	P_G_ = 0.053 P_w_ = 0.45 P _W*G_ = 0.15
Monodehydroascorbate reductase (MDHAR)	0.37 ± 0.08	0.26 ± 0.13	0.05 ± 0.02	0.08 ± 0.04	0.47 ± 0.07	0.26 ± 0.04	P_G_ < 0.001 P_W_ = 0.11 P _W*G_ = 0.27
Abscisic acid (ng/g FW)	818 ± 166.4	3403 ± 884.4	1751 ± 461.1	1057 ± 64.3	487 ± 101.6	1554 ± 399.6	P_G_ = 0.014 P_W_ = 0.002 P _W*G_ < 0.001

***WW*** Well-watered, ***D*** Drought, ***P***_***W***_ P value of drought effect, ***P***_***G***_ *P* value of genotype effect and ***P***_***WxG***_ *P* value of the interaction of drought by genotype

Neither genotypes nor drought changed the activities of DHAR, MDHAR and GR statistically. Differences were significant among genotypes for GST where, highest activity was recorded in genotype L_1_ as compared to other two genotypes. Compared to the well-watered controls, drought significantly increased the activity of GST. Likewise, the interaction of water*genotype was also significant and pronounced increase from 1.49 to 10.27 nkat g^− 1^ FW was recorded in genotype L_3_. Differences were significant for the activity of POX among genotypes, where greater activity was observed in genotype L_1_ compared to other two. However, non-significant differences for the activity of POX were recorded between the well-watered and drought-stressed plants. Similarly, differences were also significant among genotypes where, highest activity for cwPOX was observed in genotype L_3_ compared to other two genotypes. Moreover, cwPOX was significantly affected by drought (Table 4).

### Abscisic acid and antioxidants activities within spike

The ABA concentration was significant among genotypes where, highest ABA was recorded in L_1_ compared to other two genotypes. As expected, ABA concentration was significantly increased by drought in comparison to well-watered controls. There was also a significant interactive effect of water*genotype on spike ABA concentration where pronounced increase of ABA by drought was recorded in genotype L_3_ in relation to L_1_ and L_3_ (Table 4).

Differences were significant among genotypes for GST activity and the highest value was recorded in genotype L_1_ and lowest in L_3_. Drought significantly increased the activity of GST in comparison to well-watered controls. Likewise, differences were significant among genotypes for the activity of DHAR where, highest activity was recorded in L_2_ in comparison to the other two genotypes. The activity of GR was significantly different between the genotypes and the highest value was recorded in L_1_. No significant differences were observed for the activities of DHAR and GR between the well-watered and the drought stressed plants. Significant interaction of water*genotype was observed for GR where, pronounced decrease in the activity of GR under drought stress was observed in L_1_. Differences were significant among genotypes for the activity of POX and the highest value was observed in L_3_. The activity of POX was not significantly altered under drought stress (Table 4). Moreover, neither genotype nor drought significantly affected the activity of cwPOX. Differences were significant among genotypes for the activity of MDHAR where the lowest value was observed for L_2_ as compared to other genotypes. In relation to the well-watered control, drought did not affect activity of MDHAR (Table 4).

### Agronomic parameters

**Fig. 1:**
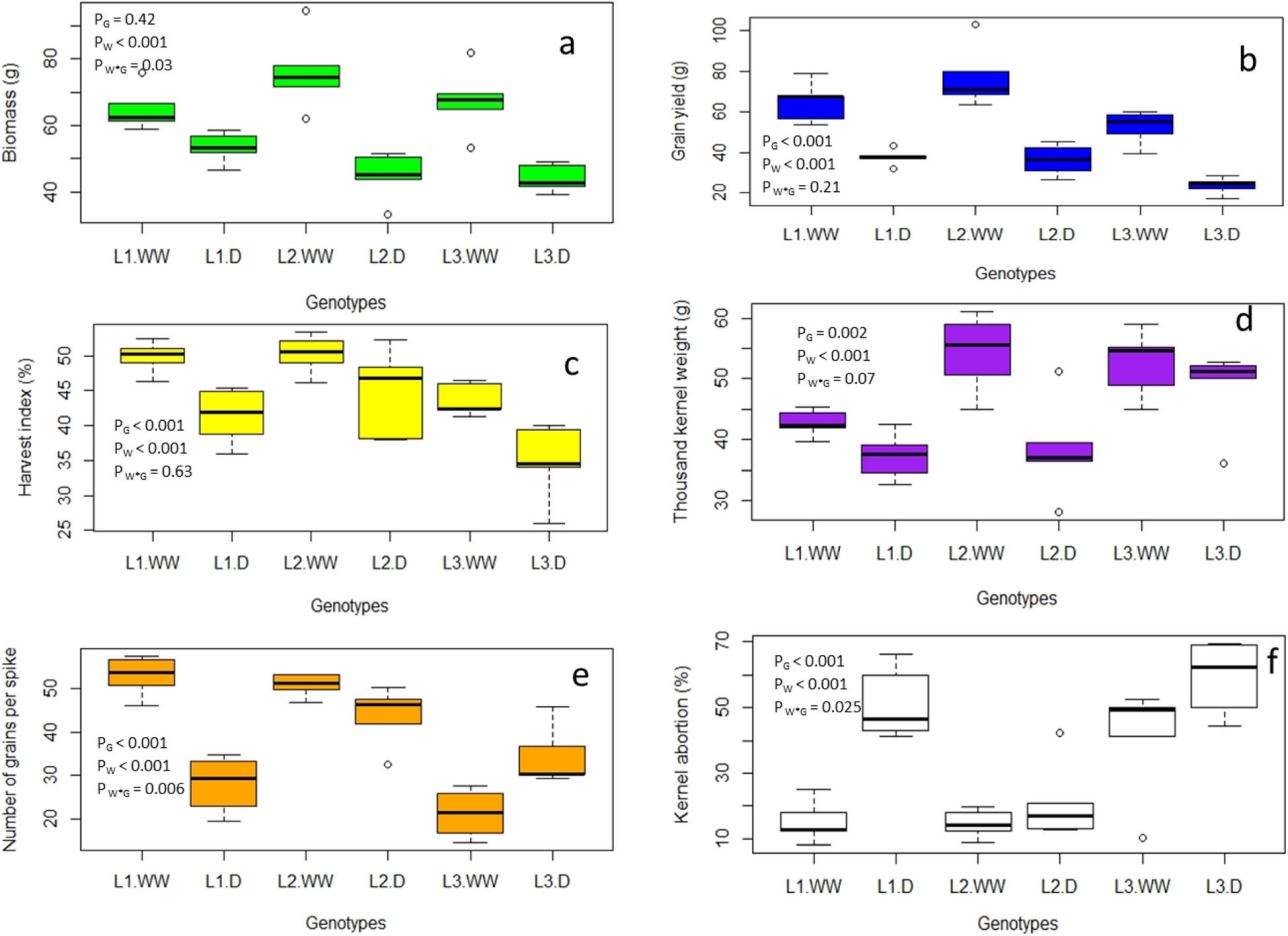
Analysis of variance for the three genotypes and boxplots for biomass (**a**) grain yield (**b**), harvest index (**c**), thousand kernel weight (**d**), number of grains spike^− 1^ (**e**) and kernel abortion (**f**) under well-watered and drought conditions. **WW** = well-watered; **D** = drought; **P**_**W**_ **=** *P* value of drought effect; **P**_**G**_ = *P* value of genotype effect and **P**_**WxG**_ = *P* value of the interaction of drought by genotype

**Fig. 2:**
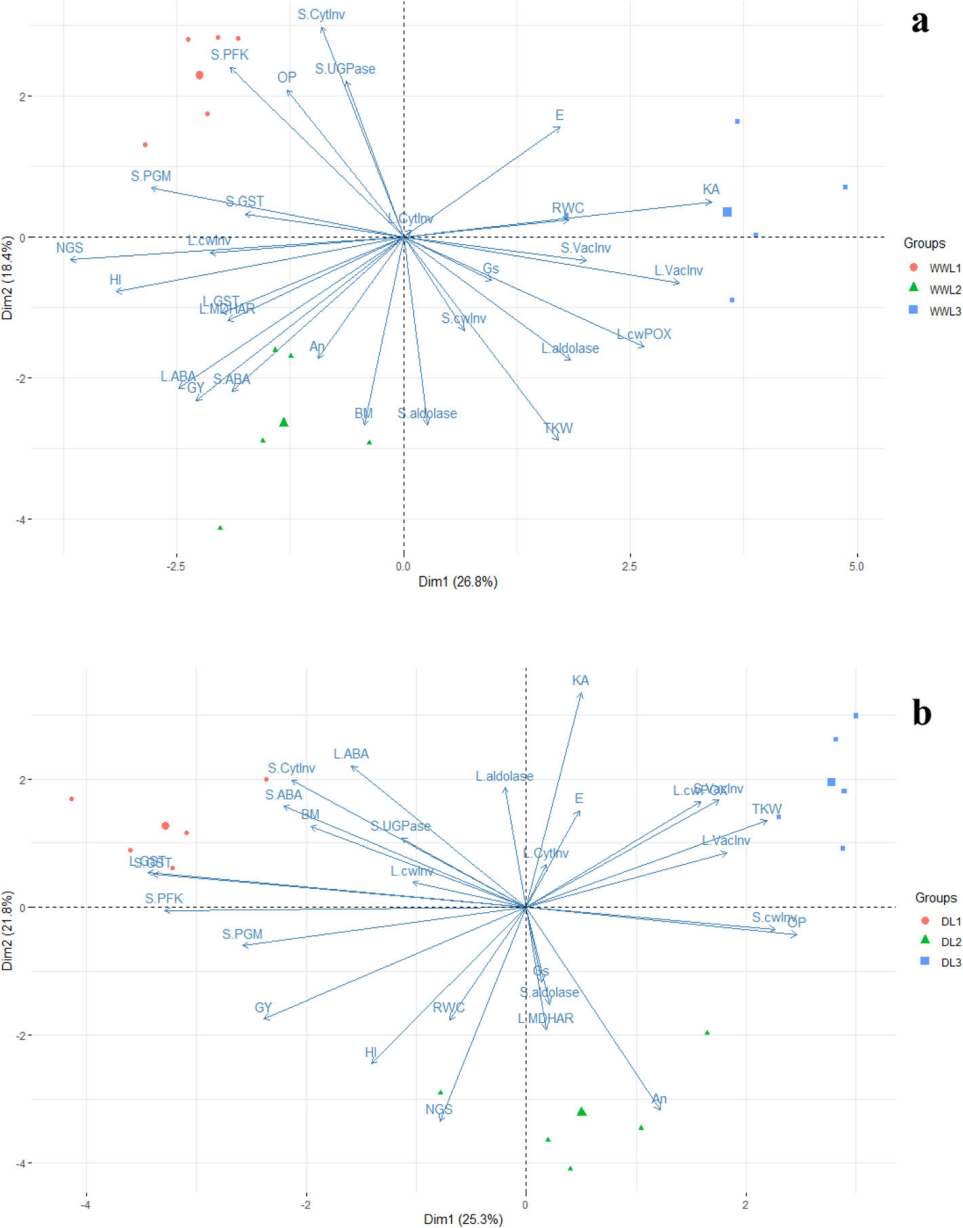
Biplot of PC1 and PC2 derived from PCA analysis under well-watered (**a**) and drought condition (**b**) Prefix “L” is indicating leaf antioxidant or carbohydrate metabolic enzymes or phytohormones and prefix “S” is indicating spike antioxidant or carbohydrate metabolic enzymes or phytohormones

Shoot biomass was identical among the three genotypes, while it was significantly reduced by drought in comparison to the control. There was significant interaction between water*genotype on shoot biomass, where more reduction in plant biomass by drought was recorded in L_2_ in comparison to the other two genotypes (Fig. 1a). Grain yield pot^− 1^ (GY) and harvest index (HI) were significantly different between the three genotypes withL_3_ having the lowest GY and HI. In comparison to the well-watered control, GY and HI were significantly reduced under drought (Fig. 2a &b).

Differences were also significant among genotypes for TKW with the highest value recorded for L_3._ Drought significantly reduced the TKW in comparison to well-watered control (Fig. 1d). The number of grains spike^− 1^ (NGS) was significantly different among three genotypes with the highest NGS recorded for L_2_ and lowest for L_3_. Moreover, in comparison to well-watered control, drought significantly reduced NGS. Additionally, significant interaction of water*genotype was recorded for NGS, where pronounced grain reduction due to drought was found in genotype L_1_ as compared to L_2_ and L_3_ (Fig. 1e). Kernel abortion (KA) was significantly different among all genotypes. Highest KA was recorded in genotype L_3_ and lowest in L_1_ (Fig. 1f). As compared to well-watered controls, drought significantly increased KA. Moreover, interaction between water*genotype was also significant and pronounced reduction was noticed in L_1_.

### Principal component analysis and combined correlations between yield traits and enzymatic activities

Separated PCA analyses for plants grown under well-watered and drought-stressed conditions were performed visualizing the associations between the yield traits and the enzymatic activities. Principal component 1 (Dim1) and principal component 2 (Dim2) described 26.8 and 18.4% variability among the variables for the well-watered treatment, respectively. Biplot analysis of Dim1 and Dim2 showed that cluster of NGS, GY and HI was closer to An, activity of L.cwInv and L.MDHAR, and these variables were in opposite direction of the cluster for L.vacInv, S.vacInv, and L.cwPOX. The activities of S and L.aldolase clustered closer to BM and in opposite direction to S-cytInv (Fig. 2a). Under drought, 25.3 and 21.8% of variability was described by PC_1_ and PC_2_, respectively (Fig. 2b). Biplot of these PC’s showed that NGS, RWC, HI and GY were clustered closer to An, Gs, S.aldolase and L.MDHAR and were in opposite direction of L.vacInv and S.vacInv, KA, TKW and L.cwPOX.

#### Combined correlation of leaf parameters with yield-related traits

A strong and positive correlation (≤ 0.56***) of ABA was recorded with GST and Ψ_π_, it was moderate (≤ 0.46**) with cwInv and cytInv and weak with KA (≤ 0.36**). ABA showed a strong negative correlation (≤ − 0.56***) with An, Gs and E, moderately negative with NGS (≤ − 0.46***) and weakly negative (≤ − 0.36*) with aldolase, cwPOX, BM, GY and NGS (Table 5). A moderate positive correlation of cwInv was recorded with GST and Ψ_π_. It was strong and negative with TKW, moderate and negative with An, Gs and E while weak and negative with aldolase, BM and GY. CytInv showed a weak and negative correlation with An. VacInv has a weak positive correlation with Ψ_π_, moderately negative with HI and weakly negative with GY and NGS. A strong positive correlation of aldolase was estimated with cwPOX, An, Gs, E, BM and TKW and it was moderate and positive with GY while the correlation of aldolase was strong and negative with GST and Ψ_π_.

**Table 5:**
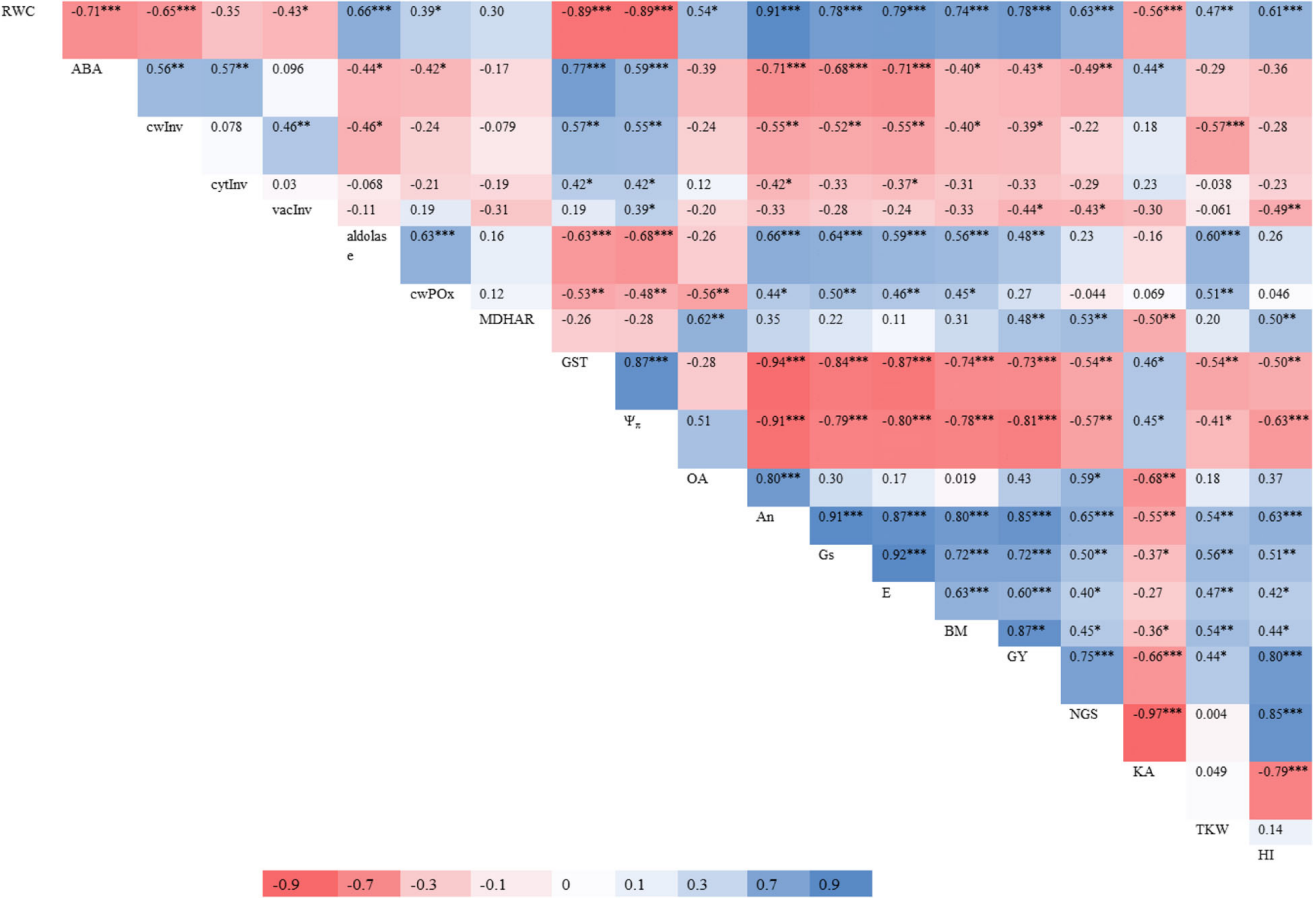
Combined correlations of leaf carbohydrate metabolic and antioxidants activity with leaf water relations, gaseous exchange, abscisic acid, and with yield and yield contributing traits

*RWC* relative water content, *ABA* Abscisic acid, *cwInv* Cell wall invertase, *cytInv* Cytoplasmic invertase, *vacInv* Vacuolar invertase, *cwPOX* Cell wall peroxidase, *MDHAR* Monodehydroascorbate reductase, *GST* Glutathione- S-transferase, *Ψ*_*π*_ Leaf osmotic potential, *OA* Osmotic adjustment, *An* Photosynthesis, *Gs* Stomatal conductance, *E* Transpiration rate, *BM* Plant biomass, *GY* Grain yield, *NGS* Number of grains spike^-1^, *KA* Kernel Abortion, *TKW* Thousand kernel weight and *HI* Harvest index

P* < 0.05, P** < 0.01 and P*** < 0.001

Moderate positive correlation of cwPOX was measured with Gs, E and TKW and it was weakly positive with An and BM. MDHAR showed moderate and positive correlation with OA, NGS, GY and HI. The correlation of GST was strong and negative with An, Gs, E, BM and GY, moderate and negative with NGS, TKW and HI. Negative Ψ_π_ showed negative correlation with most of the yield related traits except KA. However, this correlation was strong with An, Gs, E, BM, GY and HI while it was moderate and weak with NGS and TKW, respectively. A strong and positive correlation of OA was estimated with An while it was weak and positive with NGS.

A strong positive correlation of An was noticed with most of yield related traits except TKW where it was moderately positive. Correlation of Gs was similar to An except for NGS, TKW and HI where it was moderate and positive. The correlations of An and Gs were negative with KA. E was showed weak and positive with NGS and HI. A strong and positive correlation of GY recorded with NGS and HI however, correlation was weak and positive with TKW. Like GY, correlation of NGS was strong and positive with HI. In contrast, KA showed strong and negative correlation with GY, NGS and HI (Table 5).

#### Correlation of spike parameters with yield related traits

A strong and positive correlation of ABA was recorded with activities of GST, it was moderate and positive with activities of PFK and weak and positive with An. Correlation of ABA was moderate and negative with Gs and weak but negative with E CwInv showed strong and positive correlation with vacInv. A strong positive correlation of vacInv was estimated with UGPase and PFK, moderate and positive with GST and weak and positive with PGM. CytInv showed weak and negative correlation with aldolase, An, BM GY and TKW. The correlation of vacInv was weak and positive with KA and correlation weak and negative with PGM, BM, GY and HI (Table 6).

**Table 6:**
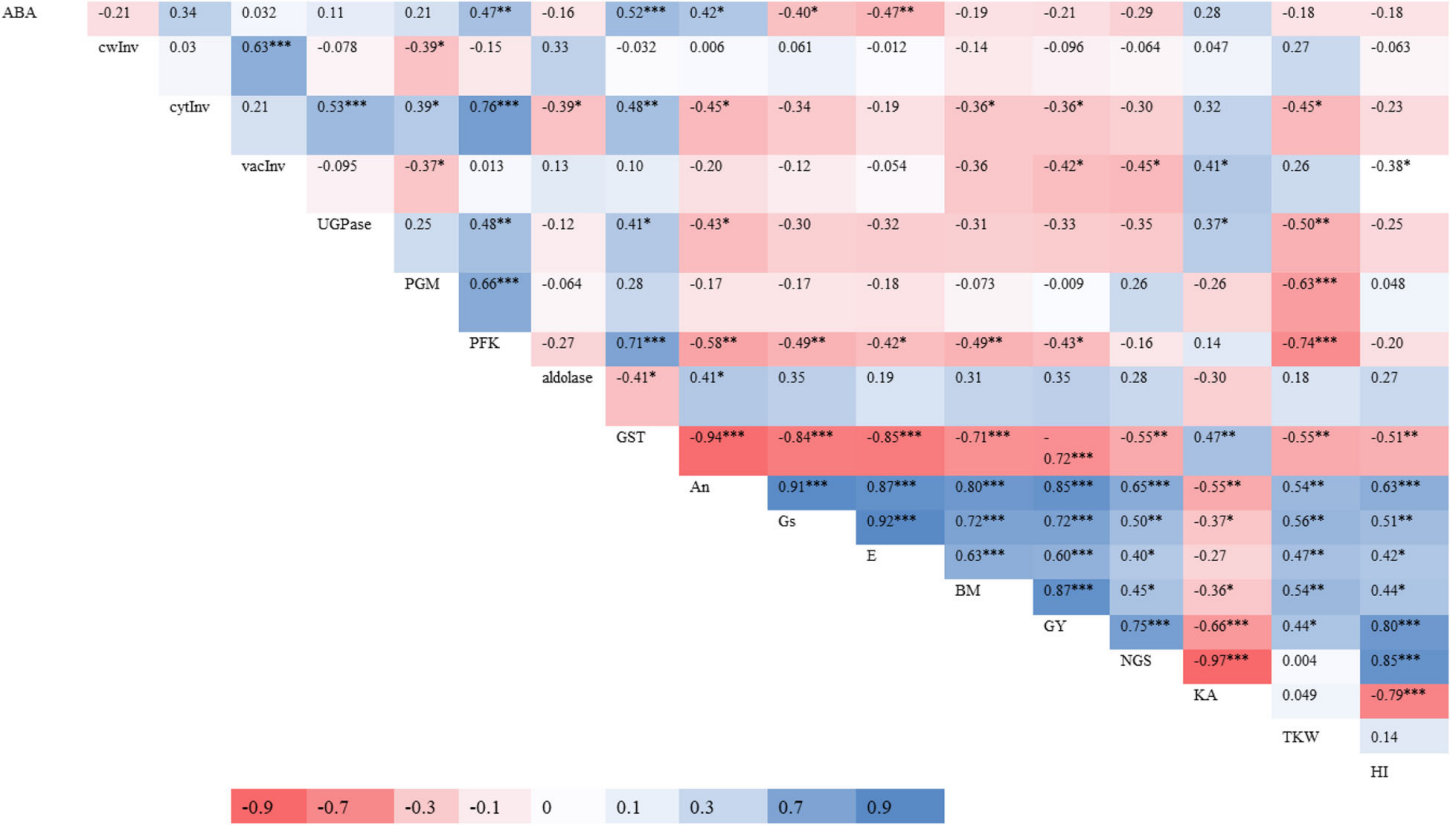
Combined correlations of spike carbohydrate metabolic and antioxidants activity with gaseous exchange, abscisic acid, and with yield and yield contributing traits

P* < 0.05, P** < 0.01 and P*** < 0.001

*ABA* Abscisic acid, *cwinv* Cell wall invertase, *cytInv* Cytoplasmic invertase, *vacInv* Vacuolar invertase, *UGPase* UDP glucose phosphorylase, *PGM* Phosphoglucomutase, *PFK* Phosphofructokinase, *GST* glutathione- S-transferase, *An* Photosynthesis, *Gs* Stomatal conductance, *E* Transpiration rate, *BM* Plant biomass, *GY* Grain yield, *NGS* Number of grains spike^-1^, *KA* Kernel Abortion, *TKW* Thousand kernel weight and *HI* Harvest index

UGPase showed moderate and negative with TKW and weak and negative with An. A strong negative correlation of PGM and PFK was estimated with TKW. PFK also showed strong and negative with TKW moderate and negative with An, Gs and BM and weak and negative with E and GY. Aldolase showed weak and positive correlation with An and weak but negative with GST. A strong and negative correlation of GST was estimated with An, Gs, E, BM and GY and moderate negative with NGS, TKW and HI.

## Discussion

A better understanding of the physiological and biochemical mechanisms attributing to grain yield losses can be tracked by studying diverse genotypes having varying yield potential under both well-watered and drought-stressed conditions. In this study, three contrasting genotypes (L_1_, L_2_ and L_3_) having different yield potential in field were selected. Genotype L_1_ and L_3_ were selected as drought tolerant and drought sensitive, respectively, while L_2_ was of moderately tolerant (intermediate yield potential based upon their performance in the field).

Drought stress at anthesis causes limited availability of photosynthates which modifies sink capacity [39] and reduces plant biomass, yield and ultimately the harvest index [40]. The reduction of grain yield in wheat caused by “anthesis drought” was attributed to both reduced grain number and individual grain weight [41, 42]. Besides, modification of key carbohydrate-metabolic enzymes both in leaf and spike could have been associated with the reduced source activity and sink strength which resulted in increased kernel abortion and lowered thousand kernel weight [43]. The limited supply of carbohydrates and alteration in the activity of key carbohydrates metabolic enzymes may induce further modifications within the plants. Moreover, the production of reactive oxygen species (ROS) and detoxification of ROS through antioxidants is one of the most prominent mechanisms of plants response to drought stress [33].

### The correlation of an and OA with HI

It has been well established that drought reduces the carbon assimilation and photosynthate supply in crop plants [44]. This limited supply of concurrent photosynthate could also modify dynamics of key carbohydrate metabolism in both source to sink organs [45]. In this study, severe reduction in photosynthesis, stomatal conductance and transpiration rate were recorded under drought condition. The lowered An might have contributed to the decreased grain yield of the wheat genotypes due to source limitation. Osmotic adjustment (OA) is an important mechanism of plant adaptation under drought stress [46]. Moinuddin et al. [47] reported a positive association of OA with grain yield in wheat under drought. In contrast, our results showed a higher OA in L_2_ did not alleviate the grain yield reduction caused by drought stress. A high ability of plants to adjust osmotically under drought may help the plants survival during the stress which however, may reduce the yield as OA is often causing a metabolic cost [48]. In agreement with previous findings of higher metabolic requirement for OA, we found a strong positive correlation of An with OA (Table 5) indicating that higher An could be contributing towards higher OA. HI describes the partitioning of photosynthates into reproductive parts in terms of dry mass. Higher An and HI was recorded in L_2_ in comparison to other two genotypes (Table 1 & Fig. 1c respectively) indicating an important role of photosynthates contribution towards the HI. Consistent with this, Earl and Davis [49] reported a reduced HI due to limited supply of photosynthetic active radiation in maize under drought conditions. The relationship between An and HI was further studied through activity of carbohydrate metabolic enzymes.

### The correlation of HI with leaf ABA concentration and the activities of carbohydrate metabolic enzymes

Primarily, a decrease in the activities of carbohydrate-metabolic enzymes was recorded in leaves, and increased activities (except aldolase and invertases) were recorded in spikes under drought conditions. A positive correlation of ABA was recorded with cwInv (Table 5), and similar results were reported by Ji et al. [50] in rice peduncle, where a higher concentration of ABA and a higher activity of vacInv were recorded under drought stress conditions, indicating that a higher concentration of ABA may have a role in regulating invertase activity. Several studies reported that subcellular metabolism of carbohydrates within plastids, cytosol and vacuole are involved in stress related responses [51, 52]. A higher activity of vacInv under drought conditions was reported by Yamada et al. [19] and in line with this, we also recorded a higher activity of vacInv under drought. On the other hand, the increased activity of invertases in the leaf could result in accumulation of hexoses, which would contribute to more negative Ψ_π_ as it is the case for L_3_ in the present study (Tables 3 & 2 respectively; Table 5). Similar correlation and closer association of biplot was estimated between Ψ_π_ and cytoplasmic invertase indicating the role of stored sugars acting as osmolytes under drought conditions (Table 5 and Fig. 2a). A low HI and a high vacInv activity in leaf and; high activity of vacInv in spike were recorded in genotype L_3_ (Fig. 1c & Table 3 respectively). Likewise, highest leaf cytInv and lowest HI was recorded in the same genotype while vice versa for the others. Roitsch and González [16] reported that the activity of vacuolar invertase regulates sugars translocation into reproductive parts under drought stress conditions. It is further indicating that sucrose was being hydrolyzed in the source hereby reducing translocation into the sink causing a lowered HI. Moreover, our experiment showed that correlations of invertase isoenzymes with HI (Table 5) were negative which are in good agreement with earlier findings [53]. In conclusion, our results show that higher ABA accumulation correlates with the increased activity of invertase, which could be indicative for a function of ABA in regulating invertase activity. However, the increased invertase activities may not facilitate an increased HI in the studied genotypes. Instead, the liberated sugars are being utilized to decrease Ψ_π_ contributing to OA in the plants.

### The correlation between activities of carbohydrate metabolic enzymes and NGS

The association of NGS and TKW with the activities of key carbohydrate metabolic enzymes were further studied to elaborate the HI response. NGS were severely reduced under drought stress and highest KA was recorded in the drought sensitive genotype L_3_ and lowest in the intermediate drought responsive genotype L_2_. Reduction in grain number under drought stress has frequently been reported in earlier studies [54, 55]. Cattivelli et al. [56] reported that drought severely affects meiosis at anthesis, which directly impacts grain number and ultimately the grain yield. Simkin et al. [57] reported that grain yield can be improved significantly by increasing the photosynthetic rate/activity. Here, significant correlation of An was found with NGS and higher An and NGS were recorded in genotype L_2_ in comparison to other genotypes (Table 5). Semenov et al. [58] also reported fewer grains due to decreased photosynthesis. The correlation of NGS with the activities of key carbohydrate metabolic enzymes was studied and negative correlation and opposite association through PCA biplot was recorded with vacInv of sink (Table 6 and Fig. 2b). Higher activity of spike vacInv was recorded in genotype L_3_ and this genotype was also having the lowest NGS. Yamada et al. [19] reported abiotic stress-inducible transporter for monosaccharides in *Arabidopsis thaliana* termed as ESL1 might function coordinately with the activity of vacuolar invertase to regulate osmotic pressure by affecting the accumulation of sugar in plant cells under drought conditions. It is further indicating that limited photosynthetic rates may force plants to utilize stored carbohydrates under severe drought conditions but in our study these carbohydrates were seemingly not utilized to enhance grain number. During glycolysis, sucrose is converted into glucose and fructose by invertases. The hexoses are further phosphorylated with the help of HXK and FK respectively [59]. In the present study, a decreased activity of HXK and FK was recorded in the leaf. No supporting literature is available to explain our findings. However, Whittaker et al. [60] reported that higher activity of HXK in the leaves of *Sporobolus stapfianus* could be responsible for drought tolerance. Likewise, Fulda et al. [61] reported that SlFRK3, a protein responsible for the activity of FK was upregulated in drought tolerant plants of sunflower under water deficit conditions. Karni and Aloni [62] also reported a decreased activity of FK in anthers under heat stress. These studies although reported in different plant species and tissues yet our studies and previous literature indicate the limited transport of sugars under drought conditions. This limited availability of sugars could induce seed abortion resulting in lower grain numbers. Below, change in HI under drought was further discussed in relation to the role of key carbohydrate metabolic enzymes in grain filling.

### The correlation of carbohydrate catalyzing enzymes with TKW

Maintenance of higher TKW is necessary to produce higher grain yield under drought conditions in wheat. In the current experiment highest TKW and lowest HI was recorded in the drought sensitive genotype L_3_ under drought conditions. Biplot analysis indicates a close association of TKW with leaf aldolase. Individually, significant and positive correlations of leaf aldolase activity with TKW and closer association via PCA biplot were noticed (Table 5 and Fig. 2b). Aldolase has been reported to play a key role in physiochemical processes regulating plant development [63] and responses to abiotic stresses [64–67]. A successive decline in the specific activities of aldolase was reported under drought [23]. While, an overexpression of gene encoding leaf aldolase increased photosynthetic rate, enhanced growth and biomass production in tobacco plants [24]. In agreement with previous findings, here a higher activity of leaf aldolase and higher TKW was observed in L_3_ (Table 3 & Fig. 1d respectively). Additionally, Simkin et al. [68] reported that stimulation of sedoheptulose 1,7-bisphosphatase and fructose 1,6-bisphophate aldolase has improved photosynthetic efficiency as well as seed yield in *Arabidopsis*. Likewise, role of different intermediate enzymes i.e. UGPase, which is the key enzymes for sucrose synthesis/breakdown [69], PGM, provides intermediate products of glycolysis and PFK, can regulate the glycolysis process through allosteric inhibition [70] was also evaluated. Negative correlations of TKW with spike UGPase, PGM and PFK were also found under drought conditions (Table 6). No supporting literature is available to confirm the results of the present study however, AGPase is reported to have positive correlation with grain fillings [27, 29]. Maize and rice transgenes having Shrunken2 gene (Sh2r6hs), which encodes an altered AGPase activity showed increased the biomass and seed weight [71, 72]. Overexpression of the *TaLSU I* gene has significantly increased AGPase activity, which positively correlated with endosperm starch weight, grain number per spike and single grain weight [73], implying that the modification of the activities of these enzymes are associated with the grain filling process hereby influencing the TKW.

### The Correlation of antioxidant enzymes with HI, NGS and TKW

To understand the possible role of antioxidant enzyme activities in sustaining harvest index under drought stress, correlation analysis of antioxidant enzymes activity with HI, NGS and TKW were performed. Among the antioxidant enzymes, an increase in the activity of GST was recorded under drought conditions both in leaf and spike (Table 4). Cummins et al. [37] and Roxas et al. [38] also reported an increase in the activity of GST under oxidative stress in transgenic tobacco. Diverging from previous reports, a decrease in the activity of GST was recorded with increasing NGS and significant negative correlation of leaf and spike GST with grain yield traits (Table 5 & Fig. 1e). In contrast, a positive correlation of leaf MDHAR activity with the HI was noticed (Table 5), implying that plants possessing a higher activity of MDHAR in source tissue would maintain redox homeostasis, which may enhance the resistance of photosynthesis to drought stress thus sustain the HI. Melandri et al. [74] reported higher DHAR activity could reduce drought-induced grain yield losses in rice. In addition, the activity of leaf MDHAR was positively correlated with NGS indicating that higher activity of this antioxidant in the source could have enhanced the drought tolerance of the wheat plants in sustaining the grain number (Fig. 1e), though the underlying mechanisms remain unknown. In line with our results, Sudan et al. [75] reported an increased MDHAR expression and enzyme activity under drought stress. Likewise, Sultana et al. [76] reported that overexpression MDHAR contributes to salt stress tolerance in rice. Eltayeb et al. [77] reported overexpression of MDHAR gene in tobacco is involved in osmotic stress tolerance under drought conditions.

In addition, a positive correlation and closer biplot association of leaf cwPOX and aldolase with TKW were noticed in the present study (Table 5 and Fig. 2a). A higher activity of POX under drought was reported by Veljovic-Jovanovic et al. [78] while work of Devi et al. [33] on wheat genotypes suggested a higher POX activity under drought helps plant to sustain grain yield. Our results are in-line with previous findings supporting that higher activity of leaf cwPOX (Table 5) which may be the reason of less reduction in TKW. These finding explains that plant could sustain NGS and TKW through maintaining the higher activities of MDHAR and cwPOX.

## Conclusion

**Fig. 3:**
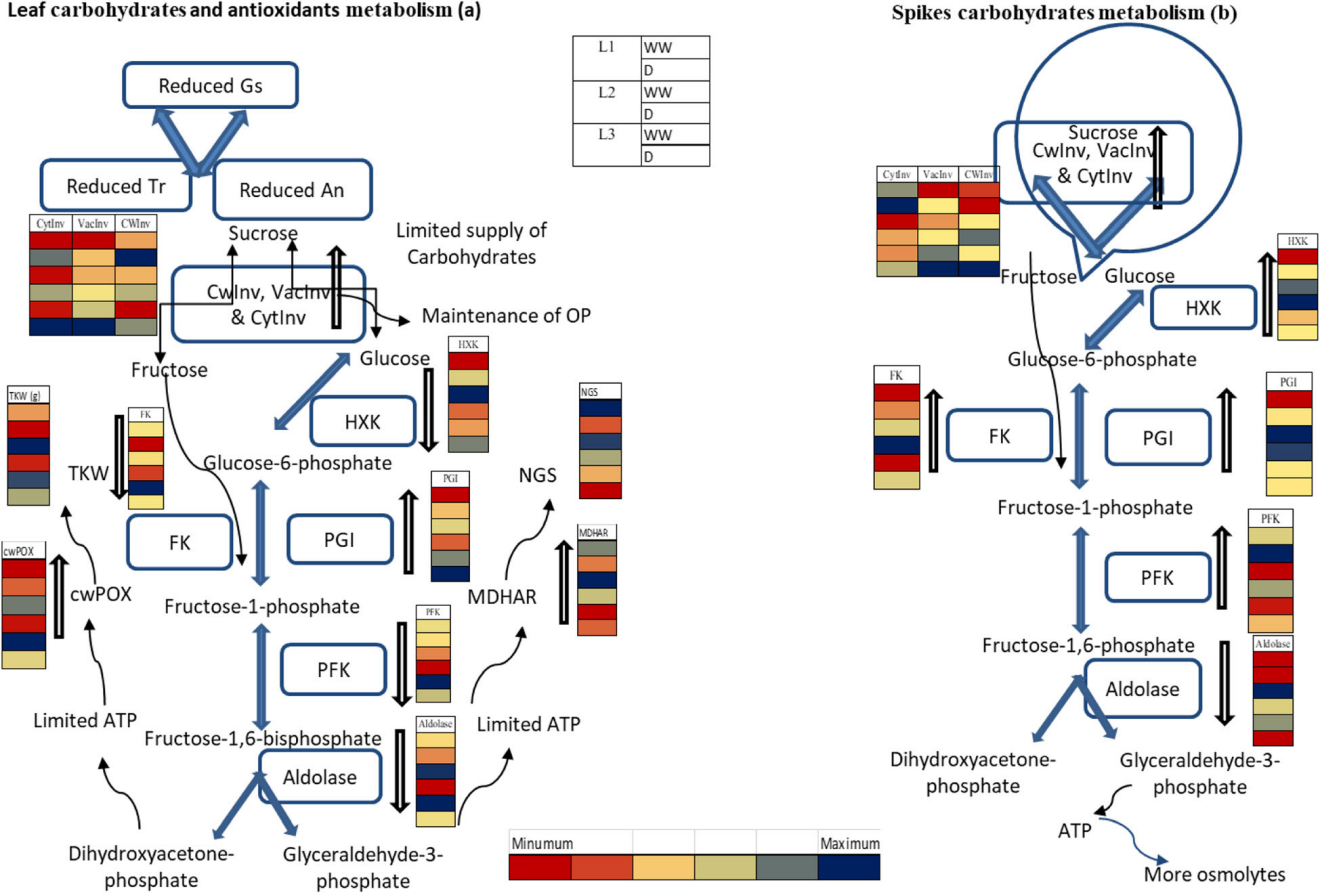
Heat map showing the effect of drought stress on carbohydrate catalyzing and antioxidant enzymes of leaf (**a**) and spike (**b**) in three wheat genotypes. Six rows of each column are indicating well-watered and drought conditions for genotype L_1_, L_2_ and L_3_, respectively. Blue color indicates highest and red color indicates lowest value respectively. All other colors represent intermediate activities/values. Small arrows are specifying conversion of sugars from on form to another. Upward bold arrows are showing rise in the activity of enzymes in comparison to well-watered conditions and downward arrows indicating decrease in the activity

Results of this study showed that drought stress at anthesis depressed photosynthesis which in turn reduced the source activity and photosynthate supply to the sink. This limited photosynthates supply could have caused reductions in NGS as well as TKW in wheat genotypes. Genotype L_1_ maintained higher grain yield both under well-watered and controlled conditions mainly due to maintenance of higher NGS, RWC and Ψ_π_ while genotype L_3_ showed less grain yield mainly due to less RWC, Ψ_π_ and higher KA under drought conditions. A high activity of aldolase, MDHAR, An, Gs and E in the source leaf might contribute towards sustaining carbohydrates remobilization from source to sink hence sustained NGS as well as HI (Fig. 3) while higher activity of aldolase and cwPOX enabled the plants to maintain a higher TKW. Under drought, a high activity of vacInv and GST in both source and sink may have contributed to enhanced production of osmolytes which is indicated by less Ψ_π_ and limited carbohydrate translocation from source and sink and higher utilization of incoming sugars by the sink may have negatively affected NGS and TKW. The findings of this study provided some insights into the biochemical mechanisms regulating grain yield of wheat in response to drought stress and distinct tolerance to drought was predicted by physiological markers which could be used as important biomarkers for breeding drought tolerant wheat cultivars for a future drier climate.

## Methods

### Plant material and growth conditions

Three genotypes [L_1_ (advanced line), L_2_ (Vorobey) and L_3_ (Punjab-11)] of contrasting drought tolerance under field conditions, developed at the International Maize and Wheat Improvement Centre (CIMMYT), Mexico and the Ayub Agricultural Research Institute (AARI) Pakistan, respectively, were selected. Genotype L_1_ and L_3_ were drought tolerant and drought sensitive, respectively, while L_2_ was of intermediate drought response (Table 7). Four seeds were sown in 4 pots of capacity liters (filled with peat material, Sphagnum, 32% organic matter, pH = 5.6–6.4 and EC = 0.45 mS cm^− 1^) and only two seedlings were remained after 1 week of emergence by thinning. Twenty-four replications for each genotype were grown under well-watered conditions.

**Table 7:** Parentage and pedigree of the analyzed wheat genotypes

Genotype	Status	Parentage	Pedigree	Centre of Origin
**L**_**1**_	Advanced Line	BCN//SORA/AE.SQUARROSA (323)/4/WBLL1/KUKUNA//TACUPETO F2001/3/BAJ #1/5/SERI.1B//KAUZ/HEVO/3/AMAD*2/4/KIRITATI	SDSS12B00908T-0Y-0B-0B-2Y-0B-0MXI	CIMMYT, Mexico [79]
**L**_**2**_	Vorobey (Approved variety)	CROC-1/AE.TA (WX-224)//OPATA-M-85/3/PASTOR[3692]	CMSS-96-Y-02555-S[3692]; CMSS-96-Y-02555-S-040Y-020 M-050SY-020SY-27 M-0Y	CIMMYT, Mexico [79]
**L**_**3**_	Punjab-11 (Approved variety)	AMSEL/ATTILA//INQ.91/PEW’S′	Pb.30196-1A-0A-2A-0A	AARI, Pakistan [80]

After few days of emergence automatic fertigation (irrigation + mixture of essential nutrients) was applied to the plants. Furthermore, weight of each pot was kept at the same water level by manual weighing of the pots. Temperatures for day/night were maintained at 22/16 °C while photoperiod was kept at 16/8 h day/night, respectively. Light conditions were maintained at 0.5 μmol photosynthetic active radiations (PAR) at night and 360 μmol PAR during the day. Likewise, relative humidity was maintained at 55 to 60%. At the time when all the plants reached to 50% flowering as described using feekes’ scale 10.3 [81], 4 replications of each genotype were harvested to study different agro-physiological parameters before applying drought stress. Remaining 20 replications of each genotype were divided into two sets: irrigation was withdrawn during anthesis for one set (10 pots) and water status of the other set (10 pots) was kept at 95% pot water holding capacity. Daily evapotranspiration (ET) of each pot was recorded by weighing. Total transpirable soil water was the change of between the pot weight at 95% water holding capacity (about 3.2 kg pot weight) and when evapotranspiration of the drought plants decreased to 10% of the well-watered plants (when pot weight was ca. 1.6 kg).

### Leaf and spikes sampling

Stress was imposed at anthesis until all the available water in the pot was consumed. In genotype L_1_ and L_2_ the drought treatment lasted for 9 days while in genotype L_3_ the drought treatment lasted 8 days. At the end of the stress period, samples were taken from both well-watered and stressed plants. Two main tillers of each plant were selected for sampling. Flag leaf and attached spike from each of the tillers were taken and snap frozen in liquid nitrogen after tightly wrapping into aluminum foil. These samples were kept at -80 °C until the further use to analyze antioxidant and carbohydrate metabolic enzyme activities and osmotic potential. Then, 5 replications of each treatment were harvested to study their eco-physiology and dry biomass of the plants (Additional file 1).

### Gaseous exchange and plant water relations

Leaf photosynthetic rate (An, μmol m^− 2^ s^− 1^) and stomatal conductance (Gs, mol m^− 2^ s^− 1^) were determined from fully expanded flag leaves between 11:00 and 14:00 h with a portable photosynthetic system (LiCor-6400XT, Li-Cor, NE, USA). Measurements were performed at 20 °C chamber temperature and 1500 μmol m^− 2^ s^− 1^ photosynthetic active radiation (PAR), and 400 ppm CO_2_ concentration in cuvette. Relative water content (RWC) were determined in flag leaves according to the method by Jensen et al. (2000) [82]. The RWC was calculated as follow:

$$ \mathrm{RWC}\left(\%\right)=\left[\left(\mathrm{FW}-\mathrm{DW}\right)/\left(\mathrm{TW}-\mathrm{DW}\right)\right]\times 100 $$

where FW and DW are leaf fresh and dry weights, respectively, and TW is leaf turgid weight.

To measure osmotic potential (Ψ_π_) of the plant tissue, frozen material wrapped in aluminum foil was thawed, squeezed, and a piece of filter paper was dipped into the obtained sap. Ψ_π_ was determined using psychrometers (C-52 sample chambers, Wescor Inc., Logan, UT, USA) connected to a datalogger (Wescor’s Dew Point Microvoltmeter, model HR-33 T). Likewise, osmotic adjustment (OA) was recorded using following formula;

$$ \mathrm{OA}=\mathrm{RWC}\left(\mathrm{well}\hbox{-} \mathrm{watered}\right)\times {\Psi}_{\uppi}\left(\mathrm{well}\hbox{-} \mathrm{watered}\right)-\mathrm{RWC}\left(\mathrm{drought}\right)\times {\Psi}_{\uppi}\left(\mathrm{drought}\right) $$

### Extraction of samples for enzymes analysis

Samples extraction was done following the protocol by Jammer et al. [30]. Briefly, leaf and 10 spikelets from the middle of the spike excluding rachis homogenized in liquid nitrogen was used. 250 mg leaf and 500 mg spike material, respectively, was extracted with 1 ml of extraction buffer consisting of 40 mM TRIS-HCl pH 7.6, 3 mM MgCl_2_, 1 mM EDTA, 0.1 mM PMSF, 1 mM benzamidine, 14.34 mM β-mercaptoethanol, 24 μM NADP and milliQ H_2_O was added into plant material to get dialyzed extract. A piece of dialysis tube for each sample (~ 3-4 cm for 1 ml sample) was cut and sealed with a clip having number on it. This setup of dialysis tube was placed in cold water (4 °C) for 15 min. Extracted supernatant was pipette into the dialysis tubes according to the arrangements. Air bubbles from the dialysis tube were removed before sealing the other end of the tube with another clip. Likewise, 1 ml of high salt buffer comprised of 1 M Tris HCl pH 7.6, 500 mM MgCl_2_, 250 mM EDTA, 4 M NaCl, ddH_2_O was added to obtain cell wall extract.

Eleven carbohydrate metabolic enzymes were selected to check their activity within leaf and spike tissue. Dialyzed extract was used for the estimation of vacInv, cytInv, AGPase, UGPase, HXK, FK, PGM, PGI, PFK, Aldolase, and cell wall extract was used to determine the activity of cwInv.

### Carbohydrate metabolic enzyme assays

Method described by Jammer et al. [30] was used to determine the activity of invertases. Concisely, 5 μl of the extract were added in flat bottom 96-well plates to determine the activity of all invertases. While, 5 μl of 100 mM sucrose and 5 μl of reaction buffer pH 4.5 (454 mM Na_2_HPO_4_/273 mM citric acid) was added into dialyzed and cell-wall extract to determine the activity of vacuolar invertase (vacInv) and cell wall invertase (cwInv) respectively while reaction buffer with pH 6.8 (772 mM Na_2_HPO_4_/114 mM Citric acid) was added into dialyzed extract to determine the activity of cytoplasmic invertase (cytInv). Sucrose was not added into control. Likewise, calibration curve was added by glucose standard (0–50 nmol). These plates were incubated at 37 °C for 30 min after adding the distilled water to raise the total reaction volume of 50 μl. Plates were put at room temperature for 20 min after removing from incubator. 200 μl of GOD-POD reagent (10 U ml^− 1^ GOD, 0.8 U ml^− 1^ POD) and 0.8 mg ml^− 1^ ABTS in 0.1 M potassium phosphate buffer, pH 7.0 was added in each well. The absorbance was measured at 405 nm of plate reader. Principle of Sung et al. [83] was used to determine the activity of all the invertase enzymes.

All remaining carbohydrate enzyme activities were determined using higher throughput method described by Jammer et al. [30]. For the activity of HXK and FK was determined following the principle of Petreikov et al. [84]. Moreover, 100 mM fructose, 50 mM NAD, 100 mM ATP, 3500 U ml^− 1^ PGI, 1000 U ml^− 1^ G_6_PDH (from *Leuconostoc mesenteroides*) and common buffer (composed of 1 M Tris HCl with pH 8.0, 0.25 M EDTA, 0.5 M MgCl_2_) was used to determine the activity of FK. TPI was not used and 100 mM fructose was replaced with 100 mM glucose to assay the activity of HXK. For the activity of UGPase and AGPase, principle of Pelleschi et al. [8] and Appeldoorn et al. [85] was used. Again, glucose and fructose were omitted from the control. For the activity of AGPase and UGPase, common buffer, 10% BSA, 100 mM Na-PPi, 10 mM NADP, 50 mM 3-PG, 1.28 U ml^− 1^ G6PDH from *Saccharomyces cerevisiae*, 1000 U ml^− 1^ PGM, 50 mM ADP-Glucose (for AGPase) and 100 mM UGP-glucose (for UGPase), was used to determine the activity of AGPase and UGPase respectively. However, for the control samples ADP-glucose and UDP-glucose were omitted. Similarly, principle of Manjunath et al. [86] was used to determine the activity of PGM. In continuation, to assay the activity of PGM 1 M Tris-HCl pH 8.0, 0.5 M MgCl_2_, 500 mM DTT, 10 mM Glc-1,6-bisP, 100 mM Glc-1-P*, 10 mM NADP, 6000 U ml^− 1^ G_6_PDH (from *S. cerevisiae*) was used. Activity of PGI was determined following the principle of Zhou and Cheng [87]. However, to determine the action PGI 10 mM glc-1,6-bisP and 100 mM glc-1-P*, were replaced with fruct-6-P* and; glc-1-P* and fruct-6-P*. Mastermix was prepared using common buffer, 25 mM fruct-1,6-bisP*, 25 mM NADH, GPDH 2100 U ml^− 1^, TPI 6000 U ml^− 1^. Additionally, activity of PFK was determined following the principle of Klotz et al. [88]. Similarly, apart from common buffer, 100 mM fruct-6-P*, 25 mM NADH, 100 mM ATP, 372 U ml^− 1^ aldolase, GPDH 2100 U ml^− 1^, TPI 6000 U ml^− 1^ was used for the activity of PFK. Fruct-6-P* was omitted as substrate in the control samples. and activity of aldolase was determined following the principle of Schwab et al. [89]. The absorbance was studied at 340 nm for 30 min and deviation of readings/peaks was monitored during this period and calculation of specific enzyme activity in nkat g FW^− 1^. Gen5 v3.04.17 software (Biotek Instruments Inc) was used to measure the absorbance of different enzymes.

### Activity of antioxidants enzymes

Methodology described by Fimognari et al. [90] was used to determine the activities of different antioxidant and 96-well plates format was utilized while, the activities were determined photometrically. Briefly, activities for ascorbate peroxidase (APX) was determined based upon the principle of Yoshimura et al. [91]. For the reactions, dialyzed extract was used. Master mix comprised of 50 mM KPO_4_ buffer pH 7.6, 0.25 mM ascorbate and 0.5 mM H_2_O_2_ was used and absorbance was recorded at 290 nm. Likewise for control H_2_O_2_ was omitted [92]. For the activities for catalase (CAT) principle of Aebi [93] was followed. Master mixed containing 50 mM KPO_4_ buffer pH 7, 0.001% antifoam agent 204 and 100 mM H_2_O_2_ was mixed with dialyzed extract and absorbance was recorded at 240 nm. Likewise, for control reactions H_2_O_2_ was omitted as mentioned by Fimognari et al. [90]. To determine the activity of peroxidase (POX) or cell wall peroxidase (cwPOX) principle of Polle et al. [94] was used. For determination of POX activity method described by Garcia-Lemos et al. [92] was used. Again, dialyzed extract was mixed with master-mix containing 100 mM KPO_4_ buffer pH 7, 2 mM guaiacol and 0.15 mM H_2_O_2_ was used. Absorbance was measured at 450 nm and H_2_O_2_ was omitted for control reactions. However, cell wall extract was used for the activity of cwPOX. The activity of superoxide dismutase (SOD) were determined following the principle of McCord and Fridovich [95]. Similarly, dialyzed extract was used to mix with master mix containing 50 mM KPO_4_ buffer pH 7.8, 0.1 mM EDTA, 0.05 mM cytochrome c, 10 mM xanthine and 0.0002 U mg^− 1^ xanthine oxidase. Absorbance was recorded at 550 nm as described by Fimognari et al. [90]. However, xanthine was omitted in control reactions. Activities of glutathione reductase (GR) principle of Edwards et al. [96] was used. Dialyzed extract was mixed with master mix containing 100 mM buffer of Tris HCl with pH 7.8, 25 mM NADPH and 30 mM glutathione oxidized (GSSG). Absorbance was detected at 340 nm for 40 min and GSSG was omitted for control reactions. For the activity of dehydroascorbate reductase (DHAR) principle of Dalton et al. [97] was followed. Again, dialyzed extract was mixed with master mix comprised of 100 mM KPO_4_ with pH 6.5, 50 mM glutathione reduced (GSH) and 50 mM dehydroascorbic acid (DHA). The activity was determined at 290 nm for 40 min and DHA was not used in control reactions. To determine the activity of monodehydroascorbate reductase (MDHAR) principle described by Arrigoni et al. [98] was followed. Dialyzed extract was mixed with reaction mixture comprised of 50 mM KPO_4_ buffer with pH 7.2, 25 mM NADH, 5 U μl^− 1^ ascorbic acid oxidase (OAA) and 50 mM ascorbate. Activity was measured at 340 nm for 40 min and ascorbate was omitted in control reactions. Additionally, the activities of glutathione S-transferase (GST) were determined following the principle of Li et al. [99]. Again dialyzed extract was mixed with reaction mixture (100 mM KPO_4_ buffer with pH 7.4, 50 mM GSH and 2,4-dinitrochlorobenzene (CDNB)). Absorbance was measured at 334 nm for 30 min and CDNB was not used for control reactions.

### Abscisic acid assay

ABA concentration in leaf and spike samples was determined through an enzyme linked immunosorbent assay (ELISA) using a monoclonal antibody for ABA (AFRC MAC252) (Asch, 2000).

### Agronomic traits measurement

At the end of drought treatment, pots from each treatment were re-watered until the maturity of the plants. Plant maturity stage was determined as described by Zadoks et al. [81]. Harvesting was done at maturity and following traits were recorded:

- Number of grains spike^− 1^ (NGS): Five spikes from each replication were taken and their averages were recorded;
- Thousand grain weight (TKW): Thousand kernel were counted from each replication and their weight was recorded in grams (g);
- Kernel abortion (KA): KA was recorded using following formula:
- (number of grains spike^− 1^ /number of florets spike^− 1^) × 100.
- Plant biomass pot^− 1^ (BM): Both plants from each pot were harvested from soil level and weight of whole plant was expressed in grams (g);
- Grain yield pot^− 1^ (GY): Spikes from the pot were threshed into grains and their weight was expressed in grams (g);
- Harvest index (HI) were recorded using following formula:Grain yield (g)/Biomass (g) × 100.

### Statistical analysis

Analysis of variance (two-way ANOVA) was done using RStudio 1.0.153 to reveal the significance of the effect of genotype, water and their interaction on the measured variables at *P* = 0.05 level. Function *PerformanceAnalytics* was used to do correlation analysis while *devtools, factoextar* and *fviz_pca_biplot* were used to do principal component analysis and to draw biplot between principal component 1 and principal component 2 “L” and “S” are indicating leaf and spike antioxidants or carbohydrate metabolic enzymes or phytohormones in biplot figure like, “L-aldolase” was used for leaf aldolase enzymes and “S-aldolase” was used for spike aldolase enzyme.

## Supplementary information

**Additional file 1.** Schematic diagram of experiment, water consumption of different genotypes during stress and sampling from different treatments and re-watering until maturity.

## Data Availability

The datasets used and/or analysed during the current study available from the corresponding author on reasonable request.

## Abbreviations

cwInv: Cell wall invertase
cytInv: Cytoplasmic invertase
vacInv: Vacuolar invertase
UGPase: UDP glucose phosphorylase
AGPase: ADP-glucose phosphorylase
HXK: Hexokinase
FK: Fructokinase
PGM: Phosphoglucomutase
PGI: Phosphoglucoisomerase
PFK: Phosphofructokinase
RWC: Relative water content
Ψ_π_: Leaf osmotic potential
NGS: Number of grains spike^− 1^
BM: Plant biomass
TKW: Thousand kernel weight
GY: Grain yield
HI: Harvest index
cwPOX: Cell wall peroxidase
MDHAR: monodehydroascorbate reductase
ABA: Abscisic Acid
GST: Glutathione- S-transferase
An: Photosynthesis
Gs: Stomatal conductance
E: Transpiration rate
