# Supplementary material for: Activities of leaf and spike carbohydrate-metabolic and antioxidant enzymes are linked with yield performance in three spring wheat genotypes grown under well-watered and drought conditions
Source: BMC Plant Biol. 2020 Aug 31;20:400. doi: 10.1186/s12870-020-02581-3 (PMC7457523; doi:10.1186/s12870-020-02581-3)
Supplement: Supplementary file 1 — Additional file 1. Schematic diagram of experiment, water consumption of different genotypes during stress and sampling from different treatments and re-watering until maturity. [file 12870_2020_2581_MOESM1_ESM.docx]

**Additional file 1.**
